# Genome-wide linkage analysis of families with primary hyperhidrosis

**DOI:** 10.1371/journal.pone.0244565

**Published:** 2020-12-30

**Authors:** Andrea B. Schote, Florian Schiel, Benedikt Schmitt, Ulrike Winnikes, Nicole Frank, Katharina Gross, Marie-Anne Croyé, Ernesto Tarragon, Adam Bekhit, Dheeraj Reddy Bobbili, Patrick May, Christoph Schick, Jobst Meyer

**Affiliations:** 1 Department of Neurobehavioral Genetics, Institute of Psychobiology, University of Trier, Trier, Germany; 2 Institute for Medical Biometry, Epidemiology and Medical Informatics, Saarland University, Homburg, Germany; 3 Bioinformatics Core, Luxembourg Centre for Systems Biomedicine, University of Luxembourg, Esch-sur-Alzette, Luxembourg; 4 Megeno, Esch-sur-Alzette, Luxembourg; 5 German Hyperhidrosis Center, Munich, Germany; German Cancer Research Center (DKFZ), GERMANY

## Abstract

Primary focal hyperhidrosis (PFH, OMIM %144110) is a genetically influenced condition characterised by excessive sweating. Prevalence varies between 1.0–6.1% in the general population, dependent on ethnicity. The aetiology of PFH remains unclear but an autosomal dominant mode of inheritance, incomplete penetrance and variable phenotypes have been reported. In our study, nine pedigrees (50 affected, 53 non-affected individuals) were included. Clinical characterisation was performed at the German Hyperhidrosis Centre, Munich, by using physiological and psychological questionnaires. Genome-wide parametric linkage analysis with GeneHunter was performed based on the Illumina genome-wide SNP arrays. Haplotypes were constructed using easyLINKAGE and visualised via HaploPainter. Whole-exome sequencing (WES) with 100x coverage in 31 selected members (24 affected, 7 non-affected) from our pedigrees was achieved by next generation sequencing. We identified four genome-wide significant loci, 1q41-1q42.3, 2p14-2p13.3, 2q21.2-2q23.3 and 15q26.3-15q26.3 for PFH. Three pedigrees map to a shared locus at 2q21.2-2q23.3, with a genome-wide significant LOD score of 3.45. The chromosomal region identified here overlaps with a locus at chromosome 2q22.1-2q31.1 reported previously. Three families support 1q41-1q42.3 (LOD = 3.69), two families share a region identical by descent at 2p14-2p13.3 (LOD = 3.15) and another two families at 15q26.3 (LOD = 3.01). Thus, our results point to considerable genetic heterogeneity. WES did not reveal any causative variants, suggesting that variants or mutations located outside the coding regions might be involved in the molecular pathogenesis of PFH. We suggest a strategy based on whole-genome or targeted next generation sequencing to identify causative genes or variants for PFH.

## Introduction

Primary focal hyperhidrosis (PFH, OMIM %144110) is a relatively common, yet poorly understood disorder. Different subtypes of PFH have been described; palmar, plantar, axillary and combinations of these are among the most frequently mentioned [1,2]. Prevalence varies, according to the ethnic composition of the sample, between 1.0% and 6.1%, with lowest prevalence in Asians and highest in White/European Americans [3–5]. It is characterised by i) excessive sweating often induced by emotional stress, ii) a strong inhibition of the quality of life including symptoms of anxiety, mild depression and social isolation, and iii) a disproportionate aggregation of the condition within families [2,3,6,7]. Although a somatic aetiology has meanwhile been accepted, neither genetic nor physiological or anatomical studies have been able to pinpoint the condition’s exact cause. Ample research suggests a Mendelian dominant mode of inheritance with a recurrence risk of up to 0.28 in the offspring of affected parents, a disease allele frequency of 5% in the general population and incomplete penetrance [1,2,8–10]. Twin studies to estimate heritability of PFH have not been published so far. Genetic studies on families with PFH are still sparse, as most research on the disorder is dealing with physiological matters or treatment methods. To date, only two groups have reported genetic linkage studies, resulting in different chromosomal loci. Higashimoto and colleagues investigated eleven families (42 affected, 40 non-affected subjects) using genome-wide polymorphic markers to identify a disease locus [8]. Three families yielded combined LOD scores of 3.08 at D14S283 and 3.16 at D14S264. The resulting minimal region covers 6 cM between D14S1070 and D14S990 on chromosome 14q11.2-14q13. However, as penetrance rates vary between 25% and 100% [1], a maximum 30 cM region from D14S261 to D14S70, based exclusively on affected individuals, is plausible as well. The authors discern locus heterogeneity and propose *NDRG2* (N-myc downstream regulated gene 2) as a potential candidate gene due to its role in neuronal development. More recently, Chen and colleagues established genetic linkage in a six-generation family (11 affected, 10 non-affected subjects) from South East China affected by PFH on chromosome 2q22.1-2q31.1 [9]. The locus they describe consists of a 31.26 Mega base pair (Mbp) region of weaker linkage (LOD score 0.772–1.142) between rs12999055 and rs4668136, and a 4.59 Mbp region of significant linkage (LOD score 2.24–3.03) between rs2683451 and rs643346. Copy number variants (CNVs) could not be associated with the phenotype, and whole-exome sequencing (WES) did not reveal any shared variants among affected subjects [9]. Summarising previous findings, an autosomal dominant mode of inheritance with possible locus heterogeneity of the disorder and a prevalence of around 3% can be expected. Therefore, our aim was to either confirm previous chromosomal regions or to identify new candidate loci for PFH. We performed a genome-wide linkage analysis (LA) and consecutive exome sequencing in nine families with PFH. We found four significant loci, one of them overlapping with the chromosomal region on chromosome 2q22.1-2q31.1 reported previously by Chen and colleagues [9].

## Results

### Characterisation of the PFH pedigrees

Within our sample of nine selected families, 51 out of 112 individuals were affected by PFH, 57 were non-affected and for four subjects the affection status could not be determined as self-reports and questionnaire data deferred. With 24 affected males and 27 affected females, no distinct sex-related differences in prevalence are apparent (Chi^2^ = 0.288, *p* = 0.591). These results are in line with the literature as no clear pattern for a sex-biased distribution of PFH is reported [4], although some studies found slightly higher prevalence in women [3,11–13].

**Fig 1:**
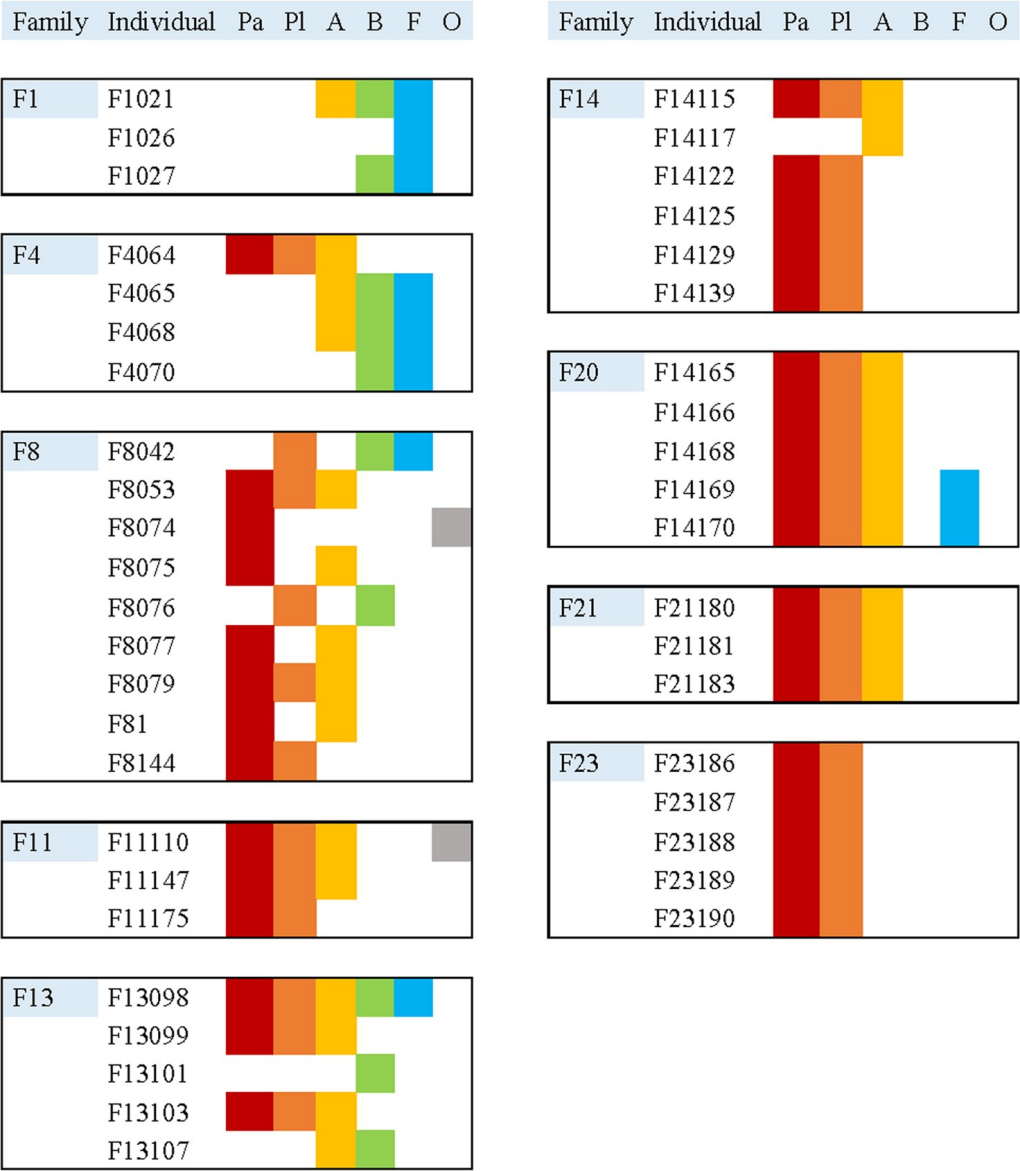
Distribution of affected body areas within families with primary focal hyperhidrosis. Only individuals with known sites were considered. A: Axillary; B: Back; F: Facial; O: Other; Pa: Palmar; Pl: Plantar.

In line with previous findings [1,2], participants in our study reported more than one affection site with expected combinations of i) palmar, plantar; ii) palmar, plantar, axillary or iii) back, facial perspiration. The most frequent subtypes of PFH with regard to affection sites were palmar hyperhidrosis with 74% and plantar hyperhidrosis with 69%. Of the affected family members, 58% reported axillary hyperhidrosis. Considerably less frequent were back and facial perspiration, which were dominant only in F1 and F4. The affected body areas showed a considerable degree of heterogeneity within most families and were homogenously distributed in only two of our families (Fig 1). Accordingly, this heterogeneity of perspiration pattern was previously shown in a Chinese family. Chen and colleagues reported that besides palms, other anatomic areas including soles and/or axillae were also affected in some of their patients [9]. The heterogeneity of affection sites in the families prevents a subtype-specific LA in our sample. This is also reflected in families F13 and F14, which have no common pattern of affected areas but share an IBD region on chromosome 2 with a significant LOD score.

### Parametric multipoint linkage analysis of nine pedigrees

Parametric multipoint LA under the assumption of autosomal dominant inheritance with all families did not reveal a genome-wide significant locus (S3 Fig), suggesting locus heterogeneity. Subsequent LA with overlapping regions of loci supported by individual families was performed to reveal additive LOD scores as described in the methods section. Four loci with a combined LOD > 3.0 were identified (Table 1 and S4 Fig), and one additional region on chromosome 2, supported by F1, F4, and F11, yielded suggestive evidence for linkage with LOD = 2.6. Of the nine families investigated for linkage, F20 did not support any of the significant or suggestive loci. Penetrance ranged from 60% in family 14 to 100% in F11, F20, and F21. Across all families contributing to one of the significant loci, 58 individuals supported the respective loci. A total of 45 of these were affected by PFH, amounting to a mean penetrance of 77.6%.

**Table 1:** Loci with genome-wide significant LOD score ≥ 3 on chromosomes 1, 2, and 15.

Chromosome	Family		Start	End	LOD
**1**	F4	bp	200,366,091	241,561,050	
		cM	196,250,000	258,490,000	
		SNP	rs12141206	rs10802960	
		G-band	1q32.1	1q43	
	F8	bp	219,001,663	235,119,382	
		cM	224,764,300	242,850,100	
		SNP	rs6541234	rs6586361	
		G-band	1q41	1q42.3	
	F23	bp	210,527,418	245,328,085	
		cM	213,910,500	268,386,000	
		SNP	rs10489388	rs12039117	
		G-band	1q32.2	1q44	
	Overlap		219,001,663	235,119,382	3.694
**2**	F1	bp	127,868,435	191,504,064	
		cM	140,090,400	190,923,100	
		SNP	rs6743470	rs1558473	
		G-band	2q14.3	2q32.2	
	F8	bp	133,639,374	164,469,186	
		cM	146,047,200	170,307,500	
		SNP	rs10928436	rs12692701	
		G-band	2q21.2	2q24.3	
	F11	bp	128,516,543	151,962,991	
		cM	140,750,500	161,460,700	
		SNP	rs7576459	rs4664951	
		G-band	2q14.3	2q23.3	
	Overlap		133,639,374	151,962,991	3.4495
**2**	F13	bp	66,815,660	100,746,935	
		cM	89,114,600	114,519,500	
		SNP	rs12987658	rs11885529	
		G-band	2p14	2q11.2	
	F14	bp	52,264,332	71,408,929	
		cM	77,289,720	94,943,840	
		SNP	rs7595947	rs12478186	
		G-band	2p16.3	2p13.3	
	Overlap		66,815,660	71,408,929	3.146
**15**	F8	bp	101,939,918	102,398,631	
		cM	132,705,300	133,756,300	
		SNP	rs1874274	rs11247329	
		G-band	15q26.3	15q26.3	
	F21	bp	93,807,754	102,398,631	
		cM	105,201,600	133,756,300	
		SNP	rs11857333	rs11247329	
		G-band	15q26.1	15q26.3	
	Overlap		101,939,918	102,398,631	3.011

bp = base pair position; cM = centimorgan position; SNP = last SNP on the inner boundary of the locus; LOD = additive Logarithm of the Odds score for all contributing loci.

### Chromosome 1, Locus 1: 1q41-1q42.3

**Fig 2:**
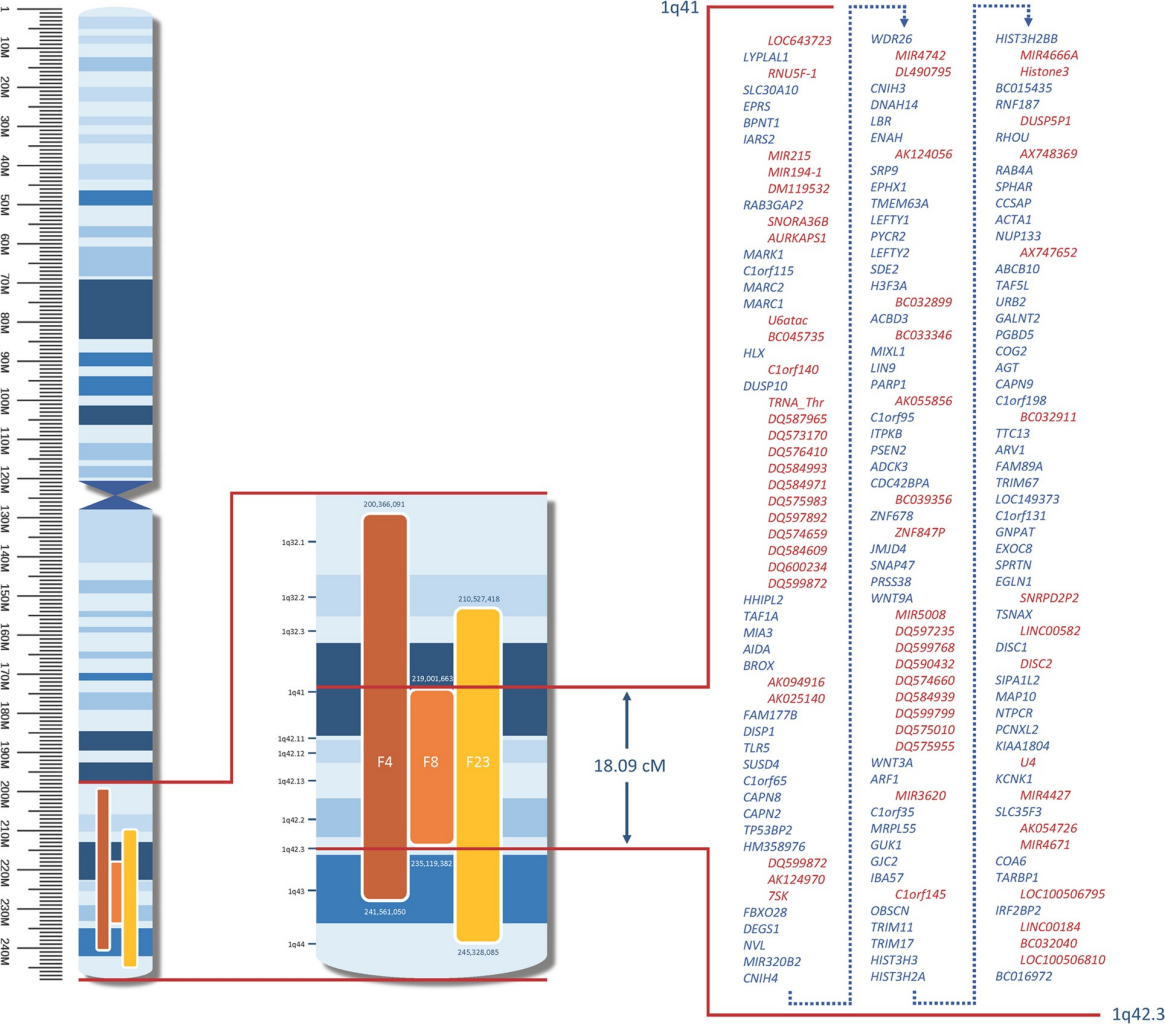
Candidate loci on chromosome 1. Coloured bars = loci supported by individual families; figures above and below these loci = physical (bp) position; bp = base pairs; M = mega base pairs; cM = centimorgan; red lines = enlarged overlap area; blue = protein-encoding genes; red = non-protein-encoding elements. All genes and positions in relation to GRCh37.p13 (hg19).

On chromosome 1, a locus spanning 18.09 cM at 1q41-1q42.3, supported by F4, F8, and F23, with a combined parametric LOD score of 3.694 at rs10737197, was identified. In this LA, 230 markers were analysed in sets of 50 markers and 0.3 cM spacing between markers. The overlapping region includes 110 protein-encoding genes and 64 non-protein-encoding elements as presented in Fig 2. The haplotypes composed of 17 markers in the candidate region at chromosome 1 are depicted in S5 Fig, which illustrates the haplotypes shared by all affected family members.

### Chromosome 2, Locus 2: 2p14-2p13.3 and Locus 3: 2q21.2-2q21.3

**Fig 3:**
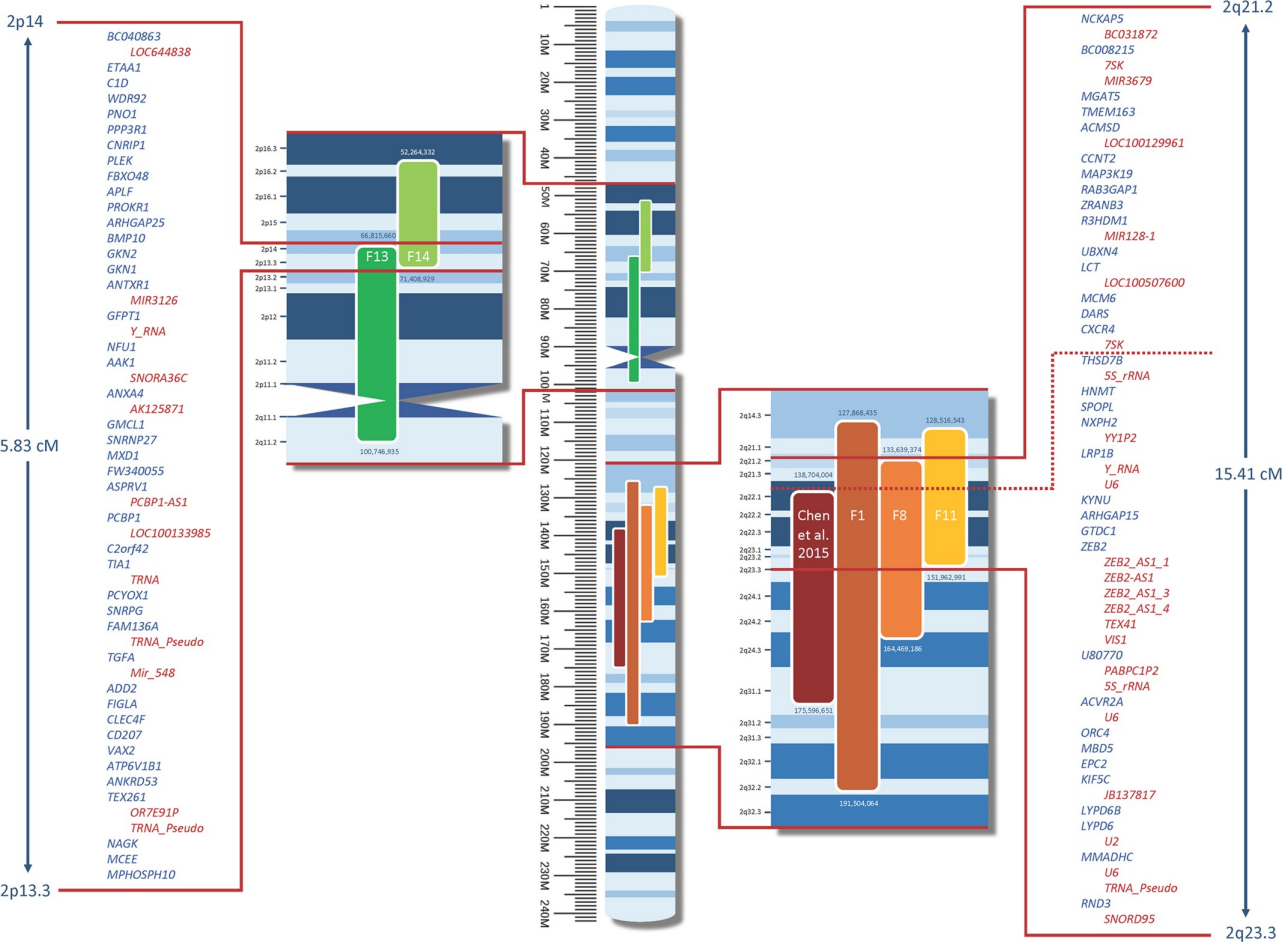
Candidate loci on chromosome 2. Coloured bars = loci supported by individual families; figures above and below these loci = physical (bp) position; bp = base pairs; M = mega base pairs; cM = centimorgan; red lines = enlarged overlap area; blue = protein-encoding genes; red = non-protein-encoding elements. All genes and positions in relation to GRCh37.p13 (hg19).

On chromosome 2, we identified one candidate locus spanning 5.83 cM at 2p14-2p13.3, supported by F13 and F14 (S6 Fig), with a LOD score of 3.146 at rs6714614, and another locus spanning 15.41 cM at 2q21.2-2q21.3, supported by F1, F8, and F11 (S7 Fig), with a LOD score of 3.45 at rs6430417 (see Fig 3). A total of 276 markers for the first locus and 321 markers for the second were analysed in sets of 50 markers with 0.3 cM spacing. The overlap between F13 and F14 includes 42 protein-encoding genes and 12 non-protein-encoding elements. Between F1, F8, and F11, we found 34 protein-encoding genes and 25 non-protein-encoding elements.

### Chromosome 15, Locus 4: 15q26.3-15q26.3

**Fig 4:**
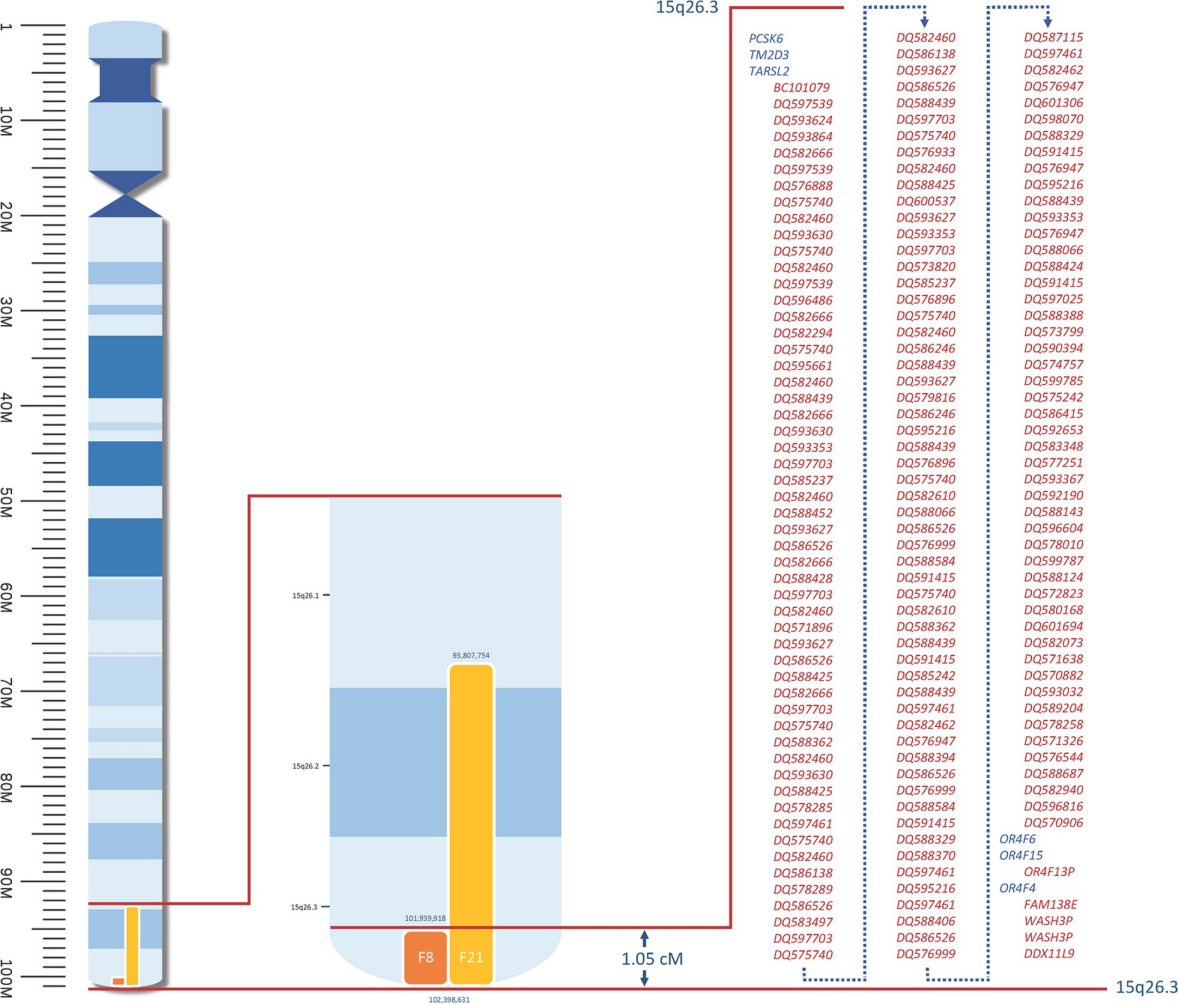
Candidate loci on chromosome 15. Coloured bars = loci supported by individual families; figures above and below these loci = physical (bp) position; bp = base pairs; M = mega base pairs; cM = centimorgan; red lines = enlarged overlap area; blue = protein-encoding genes; red = non-protein-encoding elements. All genes and positions in relation to GRCh37.p13 (hg19).

On chromosome 15, we identified a candidate locus spanning 1.05 cM at 15q26.3, supported by F8 and F21 (S8 Fig), with a LOD score of 3.011 at rs11855154 (see Fig 4). In this calculation, 184 markers in sets of 50 markers and 0.002 cM spacing were analysed. The overlap between the two families includes six protein-encoding genes and 165 non-protein-encoding elements.

The genes located at the candidate loci were tested in a functional gene enrichment analysis using gProfiler (e98_eg45_p14_ce5b097) with g:SCS multiple testing correction method applying a significance threshold of 0.05 [14]. None of the gene sets in our IBD regions are enriched for the terms relevant for hyperhidrosis “HP:0000975” or “HP:0007410”, or share a gene ontology (GO) term for “GO:biological processes”.

### WES results

The analysis of our exome-wide sequencing data revealed no variants exclusively shared by all affected members. Under the assumption of a Mendelian dominant model, the candidate loci that resulted from the LA were further screened for exonic variants as described in the methods section. This procedure revealed 64 variants at the locus on chromosome 1, 25 variants at each locus on chromosome 2 and six variants at the locus on chromosome 15. Functional gene enrichment analysis using gProfiler (e98_eg45_p14_ce5b097) with g:SCS multiple testing correction method, applying a significance threshold of 0.05 [14], did not point to a significant overexpression of any genes with human phenotype ontology terms “hyperhidrosis” (HP:0000975 or HP:0007410), or of the gene ontology terms “molecular function”, “cellular component”, or “biological process” in the database. Additional investigation of tissue-specific gene expressions, obtaining data from the GTEx Portal on 020420, showed that in total 53 of the genes in the IBD regions were expressed in brain and 46 genes in skin (Table 2). However, none of these variants/genes remained, if considering exclusively affected members of given families as carriers of the rare allele, which was a requirement of our analyses.

**Table 2:** Overview about the filtering process of whole exome sequencing data in the regions identified via linkage analysis.

Filter criteria	Variants	Genes	GTeX skin expressed	GTeX brain expressed
Total variants	369961			
MAF ≤ 5% (or NA)	71180			
Without synonymous SNVs	62840			
Without intergenic, intronic, ncRNA_intronic	19392			
CADD ≥ 15 (or NA)	14520			
GERP ≥ 3 (or NA)	13026			
Variants exclusively in affected participants	0	/	/	/
**Chromosome 1**	1329			
Candidate locus 1 (219001663–235119382)	64	36	19	25
Variants in both affected members of family 4 and family 8	0	/	/	/
**Chromosome 2**	900			
Candidate locus 2 (133639374–151962991)	25	15	10	12
Variants in both affected members of family 1 and family 8 and family 11	0	/	/	/
**Chromosome 2**	900			
Candidate locus 3 (66815660–71408929)	25	21	15	14
Variants in both affected members of family 13 and family 14	0	/	/	/
**Chromosome 15**	481			
Candidate locus 3 (101939918–102398631)	6	4	2	2
Variants in both affected members of family 8 (no data for family 21)	0	/	/	/

## Discussion

Primary focal hyperhidrosis, which is characterised by excessive perspiration of the eccrine sweat glands in palms, soles, and axillae is a heritable disorder. However, genetic analyses are sparse, and the disorder is particularly under-researched with respect to its underlying biological mechanisms. In our genome-wide LA, we investigated nine families and identified four significant loci, 1q41-1q42.3, 2p14-2p13.3, 2q21.2-2q23.3 and 15q26.3 for PFH. Our results provide additional evidence for autosomal dominant inheritance with variable penetrance and locus heterogeneity. Gene-enrichment analyses with HPO terms for hyperhidrosis and gene ontology terms for biological processes did not reveal locus-specific enrichment. Furthermore, no causative variants in exonic regions could be identified.

Primary focal hyperhidrosis is a phenotype which includes not only excessive sweating in the absence of comorbid disorders such as thyroid disease, diabetes or cancer but also an increased emotional burden [15]. Therefore, bioinformatical analyses (gProfiler, STRING, GTeX) that focus mainly on the single symptom of excessive sweating are insufficient. Using more appropriate terms such as the HPO term “palmoplantar hyperhidrosis” (https://hpo.jax.org/app/browse/term/HP:0007410) for e. g. enrichment analyses showed that no associated genes are proposed. Consequently, we exceeded the bioinformatical analyses with a manual analysis of genes and pathways suggested to be involved in emotional regulation and the sympathetic activation of eccrine sweat glands.

The cholinergic pathway plays a vital role in the regulation of thermoregulatory and emotional sweating, and is discussed as one neurotransmitter system involved in the aetiology of PFH [16]. Botulinum toxins acting as ACh release inhibitors [17] or glycopyrrolate preventing activation of fluid secretion by competitively binding to muscarinic ACh receptors [18], inhibit cholinergic pathways and provide treatment options for PFH. Besides muscarinic acetylcholine (ACh) receptors that predominantly activate eccrine sweat cells, nicotinergic ACh receptor activation plays a role as well. De Moura Júnior and colleagues reported higher expression of alpha-7 nicotinic receptor subunit (*CHRNA7*) in sympathetic ganglia of hyperhidrosis patients [19]. In addition to cholinergic innervation, noradrenergic sympathetic vasoconstrictor nerves innervate sweat glands on palms and soles [20]. In response to a stressful stimulus, this leads to sweating and sympathetic vasodilation simultaneously [21]. The relationship between hyperhidrosis and a hyperactivity of the sympathetic nervous system, demonstrated by measuring increased catecholamine levels in plasma of patients with primary palmar hyperhidrosis, suggests overactivity of the upper dorsal ganglia [22]. This is further supported by the effectiveness of sympathicolytics such as clonidine, an alpha2-adrenergic agonist [23], and endoscopic thoracic sympathectomy (ETS), in which the upper thoracic sympathetic ganglia Th2-3 are resected [24]. Although a variety of frequently unsatisfactory therapies exist, the exact mechanisms behind the effects and their interplay with corresponding genes are still under debate or even unknown.

Across all four chromosomal candidate regions identified here by IBD mapping, neither bioinformatical analyses, nor analysis of WES data revealed a strong candidate gene for hyperhidrosis. Therefore, we discuss genes of interest regarding their potential physiological relevance. Two genes, one on chromosome 1 (*PSEN2*) and the other one on chromosome 2 (*DARS*), which might be involved in the regulation of the above-mentioned cholinergic innervation of sweat glands, are located at two distinct candidate loci. *PSEN2* (presenilin 2) at 1q42.13 encodes a Ca^2+^ leak channel involved in passive Ca^2+^ efflux from the endoplasmic reticulum to the cytosol [25]. It is associated with BCHE (butyrylcholinesterase), which is vital to ACh hydrolysis, and whose corresponding gene has been suggested as “interesting” with regard to hyperhidrosis [26], although no specific interactions are documented. *DARS* (Aspartyl-tRNA Synthetase 1) at 2q21.2-2q21.3 encodes a member of a multienzyme complex that mediates the attachment of the amino acid L-aspartate to its cognate tRNAs. *DARS* together with *BCHE* were among 344 downregulated genes in tissue samples investigated for bladder cancer [27] pointing to a common interrelation of both genes. Therefore, *PSEN2* and *DARS* represent two brain-expressed genes that might be involved via their interaction with *BCHE* in the cholinergic pathway of PFH.

The candidate locus 2q22.1-2q31.1 that was reported previously [9] could be mainly supported by F1 in our study. The other two supporting families, F8 and F11, showed only weak linkage to this locus. Nevertheless, if considering our three families and the large family of Chen and colleagues [9], a minimal IBD region between 2q22.1 and 2q23.3 could be assumed (Fig 3). In the minimal region, two genes are enriched for GO:0030548 (acetylcholine receptor regulator activity) with a false discovery rate of 0.0121. *LYPD6* (Ly6/PLAUR domain containing 6) encodes a membrane-associated protein, which binds various subtypes of nicotinergic ACh receptors in the brain and acts as an inhibitor of cholinergic signalling [28]. Furthermore, increased Ca^2+^ currents and reinforced behaviour typically contingent on cholinergic neurotransmission were reported in mice with over-expressed *LYPD6* [29]. Its paralog *LYPD6B*, also located in this IBD region, is thought to enhance nACh receptor sensitivity, too [30].

False positive loci due to family size are a limitation of LA as they entail higher chances of producing statistically significant results. In our study, F8 with 26 individuals supports three loci, which consequently means that two of these most likely represent false positives, as–in a Mendelian setting–only one locus can harbour the disease-causing variant. A statistical calculation of false discovery rates–here, the amount of expected false positive loci given our family size–would not reveal valuable information because all loci identified would have to be considered as “promising” with respect to gene identification in subsequent studies. Alternatively, di-, trigenic or complex inheritance may be considered, where all three loci could be seen as valid with causative variants of smaller, albeit still relatively large effect sizes. Families contributing only to one locus each, although not genome-widely significant, would also feature additional disease loci, which were merely not detected due to low statistical power associated with small family size.

Given the partially problematic contribution of F8 to all but one of our genome-wide significant loci, the locus on chromosome 2 (2p14-2p13.3), which is supported only by F13 and F14, might be more promising. In this IBD region, the gene *PPP3R1* (calcineurin’s protein phosphatase 3 regulatory subunit B α) can be found. This gene encodes a Ca^2+^-dependent and calmodulin-stimulated protein phosphatase conferring Ca^2+^ sensitivity. *PPP3R1* binds to the InsP3R2 receptor (*ITPR2*) at the membrane of the endoplasmic reticulum, which facilitates the release of Ca^2+^ into the cytosol [31]. Interestingly, mutations of *ITPR2* have been associated with anhidrosis, such as critically low levels of perspiration, indicating a mechanism that could potentially be pathophysiologically meaningful for PFH aetiology. Furthermore, a recent GWAS identified a SNP (rs56089836) on chromosome 2, located upstream of *PPP1CB* (serine/threonine-protein phosphatase PP1-beta catalytic subunit), as associated with excessive sweating in a non-clinical cohort of Japanese females (*p* = 1.70 x 10^−11^; [32]). Protein phosphatase (PP1) is essential for cell division, participates in the regulation of glycogen metabolism, muscle contractility and protein synthesis. This protein is involved in the regulation of ionic conductance and long-term synaptic plasticity, which might play a role in excessive sweating, too. *PPP3R1* and *PPP1CB* are strongly intertwined in different pathways that are potentially meaningful for PFH, e.g. cellular sensing (STRING score 0.928, https://version11.string-db.org/).

A further limitation of this study might be the calculation of additive LOD scores based solely on those families supporting the same locus by haplotype segregation. All other families were excluded from the calculation, after no cross-familial locus could be determined for the whole family sample. Higashimoto and colleagues employed a similar strategy, which may be justified as follows: After an initial analysis including all families, no genome-wide LOD score > 3 could be observed [8]. One reason can be seen in the calculation of additive LOD scores, by which negative LOD scores lower the overall score, and a majority of families not supporting any given locus will always obscure few otherwise promising families. Failing to observe one inter-familiarly common variant might be explained due to one of three reasons: Firstly, the disorder is monogenic, albeit with a substantial degree of locus heterogeneity, which justifies clustering affected families–a procedure we have chosen in accordance with the few linkage studies on PFH published previously [8,9]. Secondly, causative variants are located in intronic or intergenetic regions or are copy number variations, which might be indicated by our WES data and which could be confirmed with whole-genome sequencing. Thirdly, one might discuss whether the disorder is complex in its nature, in which case a genome-wide association study (GWAS) ought to be preferred over LA.

Summarising our findings, we identified four genome-wide significant IBD regions in our families, which strengthens the evidence for locus heterogeneity. We could not identify any causative variants using whole-exome sequencing in the investigated participants with PFH, suggesting non-exonic regions or variants as potentially interesting. In a recent GWAS performed for excessive sweating in a Japanese population [32], the authors identified a SNP located on chromosome 2 between *PPP1CB* and *PLB1*. This finding together with our results especially for the locus 2p14-2p13.3 on the one hand provides evidence for a possible pathway involved in the aetiology of PFH and, on the other hand, shows the need for GWAS on PFH to replicate the previous findings.

## Material and methods

### Study approval and ethics statement

All methods were carried out in accordance with the relevant guidelines and regulations. The Ethics Committee from the Medical Faculty of the Friedrich-Alexander-University Erlangen-Nuremberg, Germany, has approved the experimental protocol in accordance with the declaration of Helsinki. All patients and participants provided a written informed consent for participation in the study.

### Hyperhidrosis pedigrees

The participants of the study were recruited by the Department of Neurobehavioural Genetics at the University of Trier between 2014 and 2018. In total, 83 hyperhidrotic index patients responded and received questionnaires as well as material for blood or saliva sampling. From 75 index patients, who sent back blood or saliva samples and questionnaires, 27 reported an assured family history and agreed to ask family members to cooperate in the study. For our linkage analysis, multiplex families with at least four affected members willing to take part in the study were employed. In total, 14 families with 68 hyperhidrotic and 74 healthy family members met this criterion (S1 Fig), of which nine were selected for further investigation based on family size and number of generations. Three families (F8, F13, F14) with 57 family members (affected: 23, non-affected: 32, unknown: 2) were analysed in the first round (2017) and in addition six families (F1, F4, F11, F20, F21, F23) with 55 family members (affected: 28, non-affected: 25, unknown: 2) in the second round (2018). The diagnosis for at least the index patients was based on assessments of dermatologists. Further criteria for the inclusion of patients were that excessive sweating i) exceeds an appropriate level, ii) occurs first before the age of 25, iii) occurs for no apparent reason, iv) occurs for at least 6 months and at least once a week, v) remits at night, and vi) shows extended family history with suggestive autosomal dominant transmission. All individuals were German, of Caucasian origin and native German speakers. Participants received detailed questionnaires regarding their health status. Three commonly used diagnostic instruments were included: The Hyperhidrosis Disease Severity Scale (HDSS; Solish et al. 2007), the Hyperhidrosis Impact Questionnaire (HHIQ; Hamm et al. 2006) and the Dermatology Life Quality Index (DLQI; Finlay & Khan 1994). The HDSS is a 4-point scale according to which the magnitude of the condition may be assessed. Patients are asked to state whether their sweating is 1) never noticeable and never interferes with their daily activities, 2) tolerable but sometimes interferes with their daily activities, 3) barely tolerable and frequently interferes with their daily activities, or 4) intolerable and always interferes with their daily activities (each numeral representing a score). Whereas scores 1 and 2 are classified as mild to moderate hyperhidrosis, individuals with a score of 3 or 4 would be graded as suffering from severe hyperhidrosis. The HHIQ is a 41-item questionnaire eliciting clinical factors, such as age of onset, familial aggregation, affected body areas and symmetry, as well as information about consultations with physicians and past treatments and their efficacy. Furthermore, daily activities including the influence of PFH on one’s professional life as well as emotional and psychological states are recorded. The DLQI covers similar aspects as the HHIQ, i.e. symptoms, emotional impact, etc., via 10 questions, referring to the time frame of the previous week, to be answered with 0) not at all, 1) a little, 2) a lot, or 3) very much (each numeral representing a score). The sum total ranges from 0 (no impairment) to 30 (maximum impairment). All three tests have been validated [33–35]. Furthermore, information about socioeconomic status, health history and demographic data were assessed by trained psychologists.

### Genotyping

Genomic DNA from whole EDTA-blood or saliva was available of 89 subjects from 14 different families. DNA was extracted from EDTA-blood following the salting out method by Miller and colleagues [36]. DNA from saliva was collected using Oragene kits (OG-500, DNA Self-Collection Kit, Genotek, Ottawa, Ontario, Canada) and isolated following the manufacturer’s instructions. 1,0 μg of genomic DNA from all available samples were genotyped either on Illumina HumanCore-24v1-0 (F8, F13, F14) or on Illumina InfiniumCore-24v1-1 (F1, F4, F11, F21, F20, F23) genome-wide SNP arrays by Macrogen Inc. (NGS), Seoul, South Korea. IlluminaHumanCore-24v1-0 contains assays for 306670 variants and InfiniumCore-24v1-1 for 307342 variants. Quality control included in the analysis package entailed removal of all markers with a call rate < 95% or any number of HapMap inconsistencies from the sample. GRCh37 was used as a reference.

### Population stratification

In order to account for population stratification and ensure that the samples in our current study are of European origin, we used the quality-controlled genotype data to perform multiple dimensional scaling (MDS) using PLINK 1.9 [37]. As a first step, we merged the study data with the 1000 Genomes data [38]. In the next steps, we chose only the biallelic autosomal SNVs concordant with hapmap [39]. Then the following filtering parameters were used: For controlling for linkage disequilibrium “—indep 50 5 2”, to account for Hardy-Weinberg equilibrium “—hwe 0.001”, to account for genotype missingness “—geno 0.03” and to account for minor allele frequency “—maf 0.05”. To identify the ethnicity of samples in the current study, the first and the second components from MDS clustering were visualized using R version 3.6.1 in a scatter plot (S2 Fig).

### Linkage analysis

Multipoint LA was performed with genome-wide SNP array data using SimWalk2 [40] and GeneHunter [41] on the EasyLinkage Plus v.5082 graphical user interface [42]. The final input data contained 254770 SNPs, which were checked for Mendelian errors using SimWalk2. Parametric LA was run using GeneHunter via EasyLinkage with dominant mode of inheritance, 80% penetrance and 3% prevalence. For the analysis, one marker every 0.2 cM was selected, which allowed the program to select the most appropriate markers according to its algorithm automatically within the set marker distance, intervals and call rate, and to disregard uninformative SNPs. Initial parametric LA performed with GeneHunter yielded LOD scores for all autosomes across all individuals in graphic form. According to Nyholt, a LOD score above three traditionally indicates significant linkage in study designs comparable with ours [43]. If, in this collective sample, any values above three had been observed, these would have been further investigated. As this was not the case (all LOD scores < 0, S3 Fig), families were examined individually under the assumption of locus heterogeneity (genocopy). Whenever an area within a family reached a positive LOD score, however low, it was considered a potential locus of interest. These loci were subsequently checked in the remaining families for overlap, i.e. a positive score coinciding with the first locus. When overlaps between familial loci were observed, the individual pedigrees and haplotypes were displayed in Haplopainter [44]. The haplotypes inherited identically by descent (IBD) could be verified, although merely with incomplete penetrance. Subsequently, the loci were subjected to fine-mapping, in which a higher marker density around the broadly defined haplotype borders from the initial analyses covered all used markers in the area and allowed for demarcation right down to the last SNP included upstream as well as downstream in the segregating haplotype block. The individual familial loci were then combined in an additional LA, employing the same SNP markers for all families to ensure additivity, to calculate a common LOD score across all overlapping families.

### Next generation sequencing

#### Library preparation and whole-exome sequencing

1,5 μg of genomic DNA from 31 selected individuals (24 affected individuals of 14 families, S1 Fig) was used for exome sequencing at Macrogen Inc. (Next Generation Sequencing). A SureSelectXT human all-exon library was prepared using the SureSelectXT Target Enrichment System for Illumina Version B.2, April 2015. Sequencing was performed on a NovaSeq 6000 platform with 100x coverage (50x on-target coverage = 6 Giga bp (Gbp)) following the Nova Seq 6000 System User Guide Document #1000000019358 v02 using the sequencing Nova Seq control software NCS v1.0.1. On average, the total number of reads was 62,717,339, the total number of sequenced base pairs 9.47 Gbp. The ratio of reads with a phred quality score above 30 was on average 91.89%.

#### Data processing

Sequencing adapters were trimmed and samples with < 30 × mean depth or < 70% total exome coverage at 20 × mean depth of coverage were excluded from further analysis. Variant calling was performed in targeted exonic intervals with 100 bp padding using the GATK best practices pipeline [45] against the GRCh37 human reference genome followed by multi-allelic variant decomposition and left normalisation. Samples were excluded from further analysis if they i) were not ethnically matched, ii) showed discrepancy with reported sex, iii) had an excess heterozygosity > 3 SD in any of the quality metrics as calculated by PLINKseq i-stats parameter. The genotypes of variants with read depth < 10 or genotype quality < 20 were set to missing. Variants were excluded if they i) failed variant quality score recalibration (VQSR) or GATK recommended hard filter, ii) showed missingness > 3%, iii) were present in repeat regions or iv) had an average read depth < 10 in all participants. The Exome Aggregation Consortium (ExAC) variants were restricted to the exonic intervals used for variant calling in this study and passed the VQSR threshold [46].

#### Variant annotation and filtering

Variants were annotated using ANNOVAR [47] version 2015 Mar 22 with RefSeq and Ensembl, Combined Annotation Dependent Depletion (CADD) scores [48], allele frequencies and dbNSFP (v3.0) annotations [46]. For rare variant analysis, we filtered out common variants from the European population. Therefore, we selected variants with a minor allele frequency < 0.05 in the European population of the 1000 genomes dataset, August 2015. Under the assumption of a Mendelian autosomal dominant inheritance with a prevalence of 3% and penetrance of 80%, we excluded intergenic, intronic, and ncRNA_intronic variants from the analysis and considered only non-synonymous, frameshift, splice site or stop codon variants with a Combined Annotation Dependent Depletion (CADD) score > 15 and a Genomic Evolutionary Rate Profiling (GERP) conservation score > 3. These variants were finally analysed in the chromosomal regions found to be significant in the parametric LA regarding the affection status of the family members.

## Supporting information

S1 Fig Pedigrees with segregating primary focal hyperhidrosis. Males are depicted as squares, females as circles. A slash through the symbol indicates that the individual is deceased. Clear symbols represent unaffected individuals, black symbols individuals with final diagnosis of hyperhidrosis, questionmark individuals with unclear affection status. ^a)^ Lables families that were evaluated fin genome-wide linkage analyses. Hashtags indicate those individuals with DNA specimen available. Stars in F1-F20 show individuals included in the whole-exome sequencing.
(PDF)

S2 Fig MDS Plots for the hyperhidrosis sample merged with 1000 genome data. The sample was merged either for A) all populations or B) European population using PLINK 1.9 and R version 3.6.1 for visualisation showed no stratification bias in our study sample.
(PDF)

S3 Fig Multipoint linkage analyses of chromosomes 1–22 over all nine families. Parametric model: prevalence 3%, penetrance 80%, dominant. No genome-wide significant LOD score resulted from the analysis, which was performed with GeneHunter (Kruglyak et al., 1996) via easyLinkage v5.082 (Lindner & Hoffmann, 2005). Markers were analysed in sets of 50 markers (red indications = incorporated SNPs; blue indications = boundaries between sets), spacing 0.2 cM between markers. pLOD = parametric LOD score; cM = centimorgan.
(PDF)

S4 Fig Multipoint linkage analyses of chromosomes 1, 2 and 15 to calculate additive LOD scores for selected families. Parametric model: Prevalence 3%, penetrance 80%, dominant. Four genome-wide significant loci were identified with the analyses, which were performed with GeneHunter (Kruglyak et al., 1996) via easyLinkage v5.082 (Lindner & Hoffmann, 2005). Chr1 (1q41-q42.3): 230 markers; Chr 2 (2p14-p13.3): 276 markers; Chr 2 (2q21.2-q21.3): 321 markers; Chr 15 (15q26.3-q26.3): 184 markers were analysed in sets of 50 markers (red indications = incorporated SNPs; blue indications = boundaries between sets), spacing 0.3 cM on Chr 1 and Chr 2 and 0.002 cM on Chr 15 between markers. pLOD = parametric LOD score; cM = centimorgan.
(PDF)

S5 Fig Haplotype segregation in F4, locus 1q32.1-1q43; F8, locus 1q41-1q42.3; F23, locus 1q32.2-1q44. 17 SNPs illustrating haplotypes shared by all affected family members (SNPs do not depict exact locus boundaries; for precise values, see Table 1). Square = male; circle = female; black = affected; clear = unaffected; grey = unknown affection status; diagonal dash = deceased; symbols in brackets = no DNA available; red bar = segregating haplotype; 1 = major allele; 2 = minor allele; 0 = no DNA; arrows = approximate boundaries of familial locus; SNP = single nucleotide polymorphism; cM = centimorgan.
(PDF)

S6 Fig Haplotype segregation in F13, locus 2p14-2q11.2; F14, locus 2p16.3-2p13.3. 20 SNPs (F13) or 14 SNPs (F14) illustrating haplotypes shared by all affected family members (SNPs do not depict exact locus boundaries; for precise values, see Table 1). Square = male; circle = female; black = affected; clear = unaffected; grey = unknown affection status; diagonal dash = deceased; symbols in brackets = no DNA available; red bar = segregating haplotype; 1 = major allele; 2 = minor allele; 0 = no DNA; arrows = approximate boundaries of familial locus; SNP = single nucleotide polymorphism; cM = centimorgan.
(PDF)

S7 Fig Haplotype segregation in F1, locus 2q14.3-2q32.2; F8, locus 2q21.2-2q24.3; F11, locus 2q14.3-2q23.3. 24 SNPs (F1), 16 SNPs (F8) or 21 SNPs (F11) illustrating haplotypes shared by all affected family members (SNPs do not depict exact locus boundaries; for precise values, see Table 1). Square = male; circle = female; black = affected; clear = unaffected; grey = unknown affection status; diagonal dash = deceased; symbols in brackets = no DNA available; red bar = segregating haplotype; 1 = major allele; 2 = minor allele; 0 = no DNA; arrows = approximate boundaries of familial locus; SNP = single nucleotide polymorphism; cM = centimorgan.
(PDF)

S8 Fig Haplotype segregation in F8, locus 15q26.3 and F21, locus 15q26.1-15q26.3. 11 SNPs (F8) or 19 SNPs (F21) illustrating haplotypes shared by all affected family members (SNPs do not depict exact locus boundaries; for precise values, see Table 1). Square = male; circle = female; black = affected; clear = unaffected; grey = unknown affection status; diagonal dash = deceased; symbols in brackets = no DNA available; red bar = segregating haplotype; 1 = major allele; 2 = minor allele; 0 = no DNA; arrows = approximate boundaries of familial locus; SNP = single nucleotide polymorphism; cM = centimorgan.
(PDF)
