# Supplementary material for: Genome-wide linkage analysis of families with primary hyperhidrosis
Source: PLoS One. 2020 Dec 30;15(12):e0244565. doi: 10.1371/journal.pone.0244565 (PMC7773265; doi:10.1371/journal.pone.0244565)
Supplement: S1 Fig — Males are depicted as squares, females as circles. A slash through the symbol indicates that the individual is deceased. Clear symbols represent unaffected individuals, black symbols individuals with final diagnosis of hyperhidrosis, questionmark individuals with unclear affection status. a) Lables families that were evaluated fin genome-wide linkage analyses. Hashtags indicate those individuals with DNA specimen available. Stars in F1-F20 show individuals included in the whole-exome sequencing. (PDF) [file pone.0244565.s001.pdf]

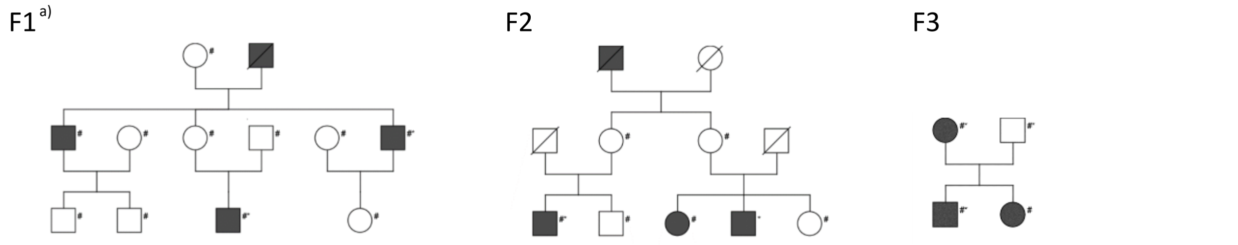

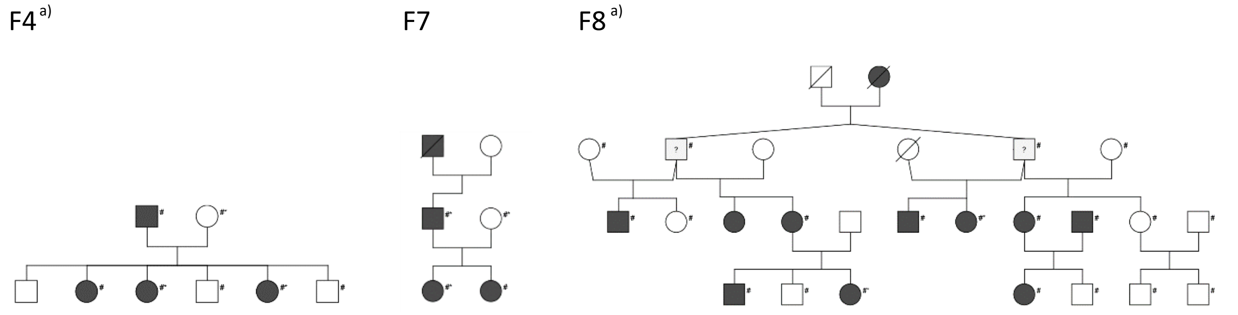

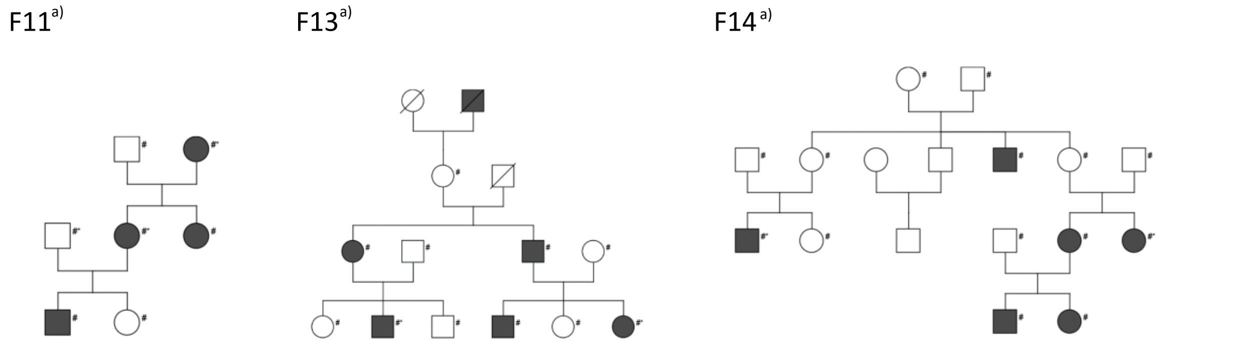

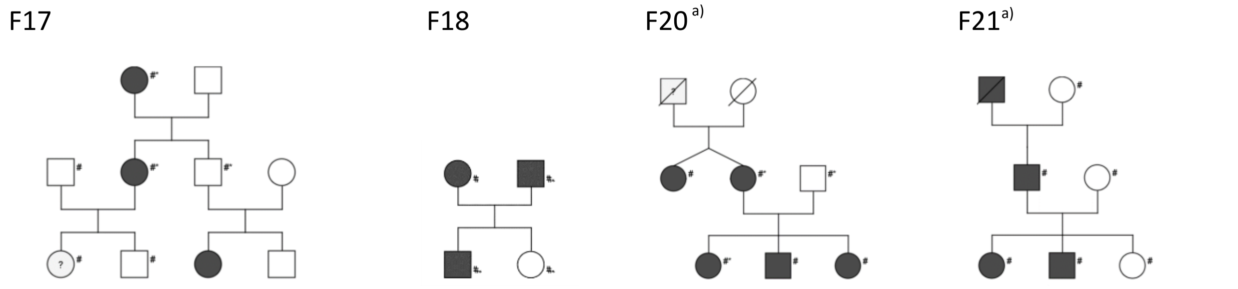

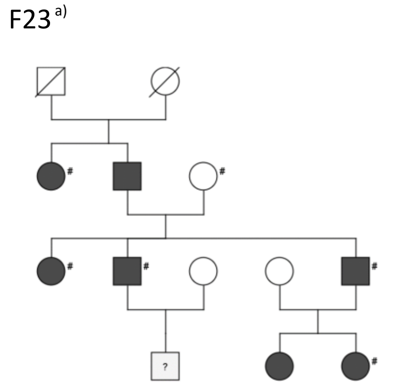

S1 Fig. Pedigrees with segregating primary focal hyperhidrosis. Males are depicted as squares, females as circles. A slash through the symbol indicates that the individual is deceased. Clear symbols represent unaffected individuals, black symbols individuals with final diagnosis of hyperhidrosis, questionmark individuals with unclear affection status. a) Lables families that were evaluated fin genome-wide linkage analyses. Hashtags indicate those individuals with DNA specimen available. Stars in F1-F20 show individuals included in the whole-exome sequencing.
