# Supplementary material for: Genome-wide linkage analysis of families with primary hyperhidrosis
Source: PLoS One. 2020 Dec 30;15(12):e0244565. doi: 10.1371/journal.pone.0244565 (PMC7773265; doi:10.1371/journal.pone.0244565)
Supplement: S2 Fig — The sample was merged either for A) all populations or B) European population using PLINK 1.9 and R version 3.6.1 for visualisation showed no stratification bias in our study sample. (PDF) [file pone.0244565.s002.pdf]

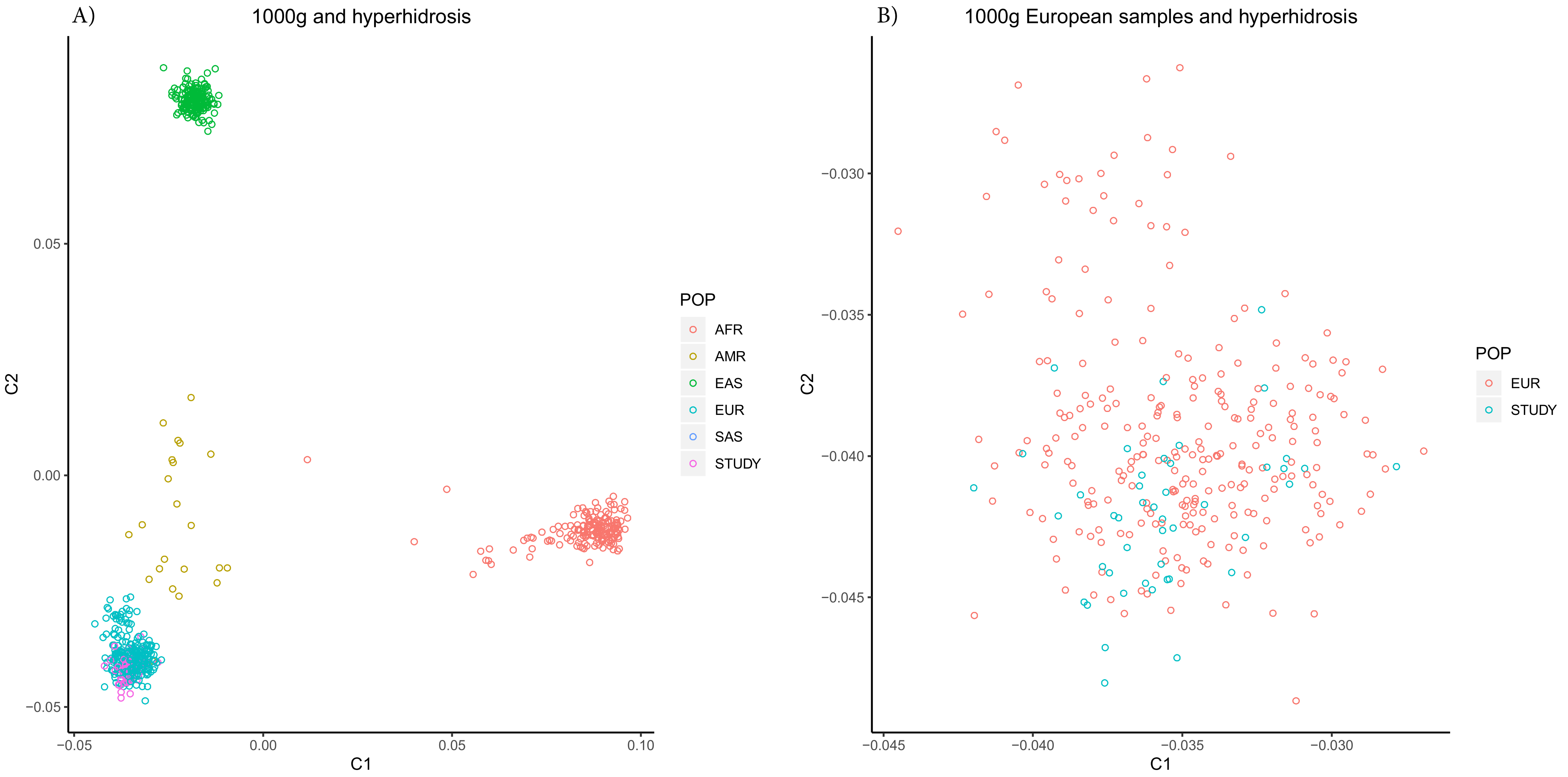

S2 Fig. MDS Plots for the hyperhidrosis sample merged with 1000 genome data. The sample was merged either for A) all populations or B) European population using PLINK 1.9 and R version 3.6.1 for visualisation showed no stratification bias in our study sample.
