# Supplementary material for: Genome-wide linkage analysis of families with primary hyperhidrosis
Source: PLoS One. 2020 Dec 30;15(12):e0244565. doi: 10.1371/journal.pone.0244565 (PMC7773265; doi:10.1371/journal.pone.0244565)
Supplement: S3 Fig — Parametric model: prevalence 3%, penetrance 80%, dominant. No genome-wide significant LOD score resulted from the analysis, which was performed with GeneHunter (Kruglyak et al., 1996) via easyLinkage v5.082 (Lindner & Hoffmann, 2005). Markers were analysed in sets of 50 markers (red indications = incorporated SNPs; blue indications = boundaries between sets), spacing 0.2 cM between markers. pLOD = parametric LOD score; cM = centimorgan. (PDF) [file pone.0244565.s003.pdf]

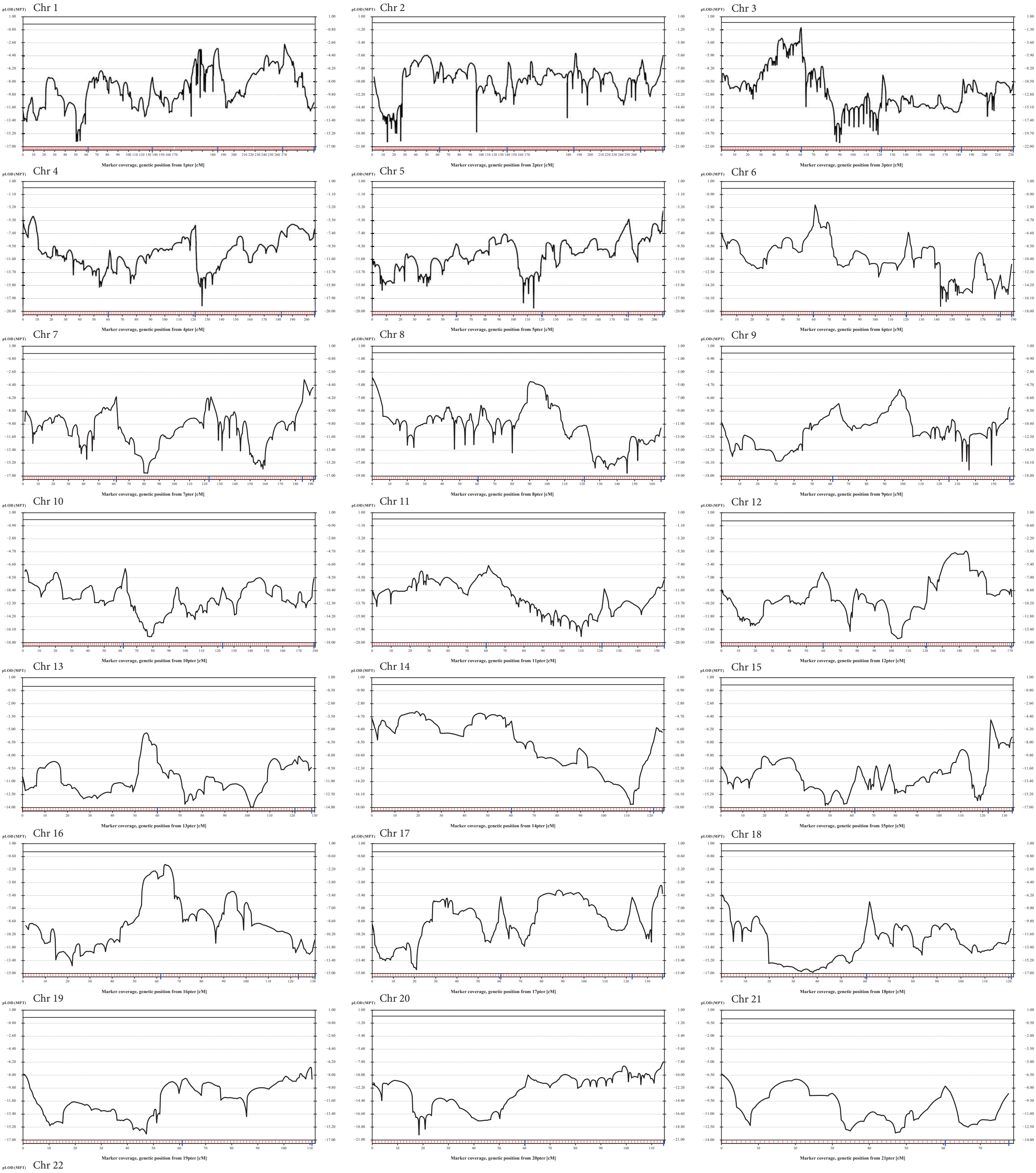

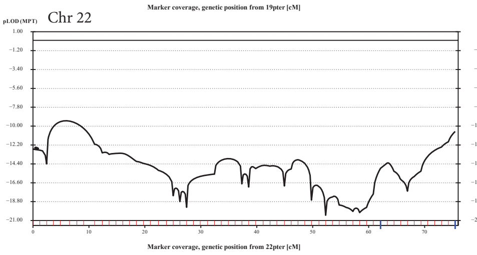

S3 Fig. Multipoint linkage analyses of chromosomes 1-22 over all nine families. Parametric model: prevalence 3 %, penetrance 80 %, dominant. No genome-wide significant LOD-Score resulted from the analysis, which was performed with GeneHunter (Kruglyak et al., 1996) via easyLinkage v5.082 (Lindner & Hoffmann, 2005). Markers were analysed in sets of 50 markers (red indications = incorporated SNPs; blue indications = boundaries between sets), spacing 0.2 cM between markers. pLOD= parametric LOD score; cM = centimorgan.
