# Supplementary material for: Genome-wide linkage analysis of families with primary hyperhidrosis
Source: PLoS One. 2020 Dec 30;15(12):e0244565. doi: 10.1371/journal.pone.0244565 (PMC7773265; doi:10.1371/journal.pone.0244565)
Supplement: S4 Fig — Parametric model: Prevalence 3%, penetrance 80%, dominant. Four genome-wide significant loci were identified with the analyses, which were performed with GeneHunter (Kruglyak et al., 1996) via easyLinkage v5.082 (Lindner & Hoffmann, 2005). Chr1 (1q41-q42.3): 230 markers; Chr 2 (2p14-p13.3): 276 markers; Chr 2 (2q21.2-q21.3): 321 markers; Chr 15 (15q26.3-q26.3): 184 markers were analysed in sets of 50 markers (red indications = incorporated SNPs; blue indications = boundaries between sets), spacing 0.3 cM on Chr 1 and Chr 2 and 0.002 cM on Chr 15 between markers. pLOD = parametric LOD score; cM = centimorgan. (PDF) [file pone.0244565.s004.pdf]

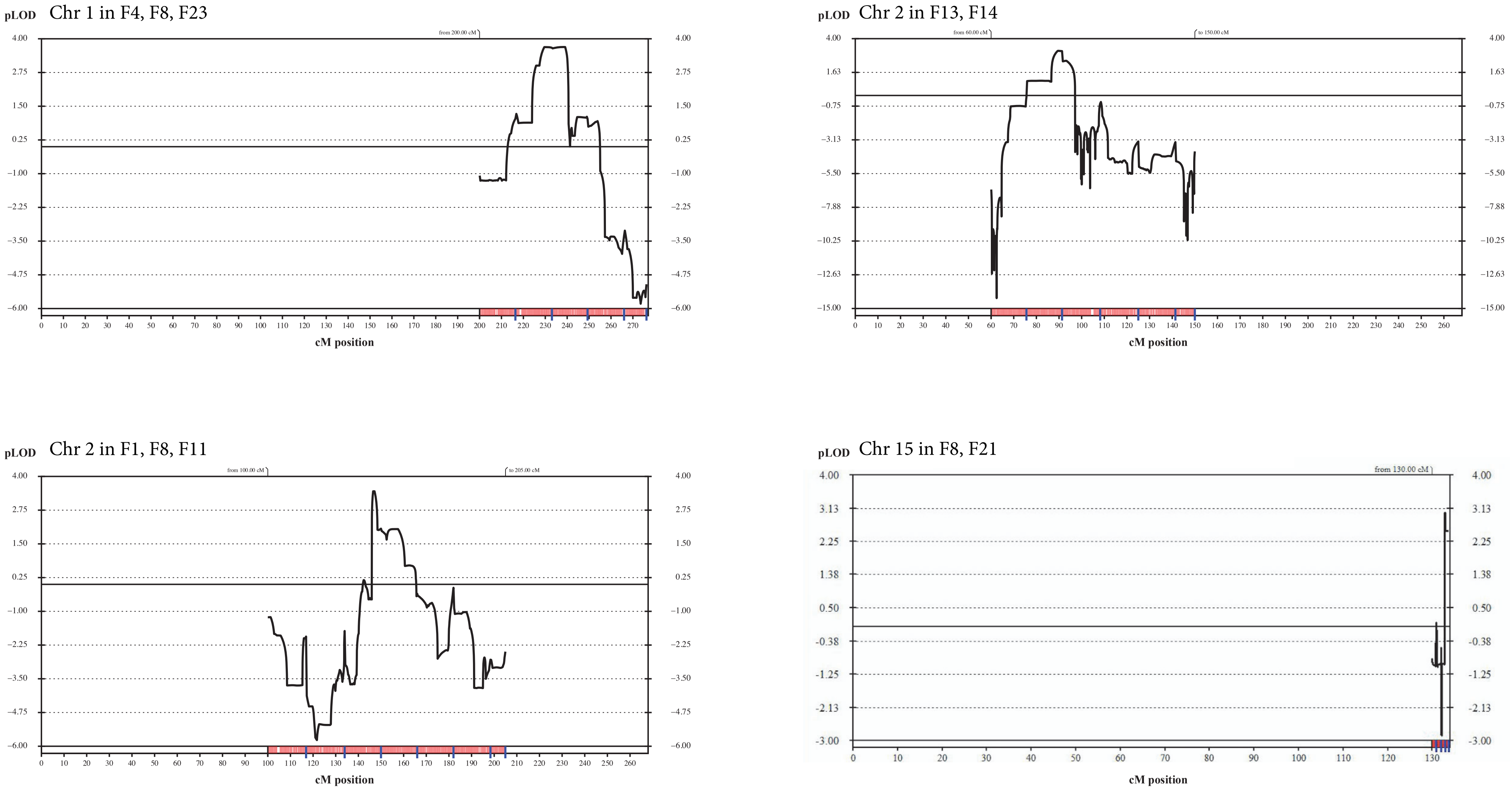

S4 Fig. Multipoint linkage analyses of chromosomes 1, 2 and 15 to calculate additive LOD scores for selected families. Parametric model: Prevalence 3 %, penetrance 80 %, dominant. Four genome-wide significant loci were identified with the analyses, which were performed with GeneHunter (Kruglyak et al., 1996) via easyLinkage v5.082 (Lindner & Hoffmann, 2005). Chr1 (1q41-q42.3): 230 markers; Chr 2 (2p14-p13.3): 276 markers; Chr 2 (2q21.2-q21.3): 321 markers; Chr 15 (15q26.3-q26.3): 184 markers were analysed in sets of 50 markers (red indications = incorporated SNPs; blue indications = boundaries between sets), spacing 0.3 cM on Chr 1 and Chr 2 and 0.002 cM on Chr 15 between markers. pLOD = parametric LOD score; cM = centimorgan.
