# Supplementary material for: Genome-wide linkage analysis of families with primary hyperhidrosis
Source: PLoS One. 2020 Dec 30;15(12):e0244565. doi: 10.1371/journal.pone.0244565 (PMC7773265; doi:10.1371/journal.pone.0244565)
Supplement: S6 Fig — 20 SNPs (F13) or 14 SNPs (F14) illustrating haplotypes shared by all affected family members (SNPs do not depict exact locus boundaries; for precise values, see Table 1). Square = male; circle = female; black = affected; clear = unaffected; grey = unknown affection status; diagonal dash = deceased; symbols in brackets = no DNA available; red bar = segregating haplotype; 1 = major allele; 2 = minor allele; 0 = no DNA; arrows = approximate boundaries of familial locus; SNP = single nucleotide polymorphism; cM = centimorgan. (PDF) [file pone.0244565.s006.pdf]

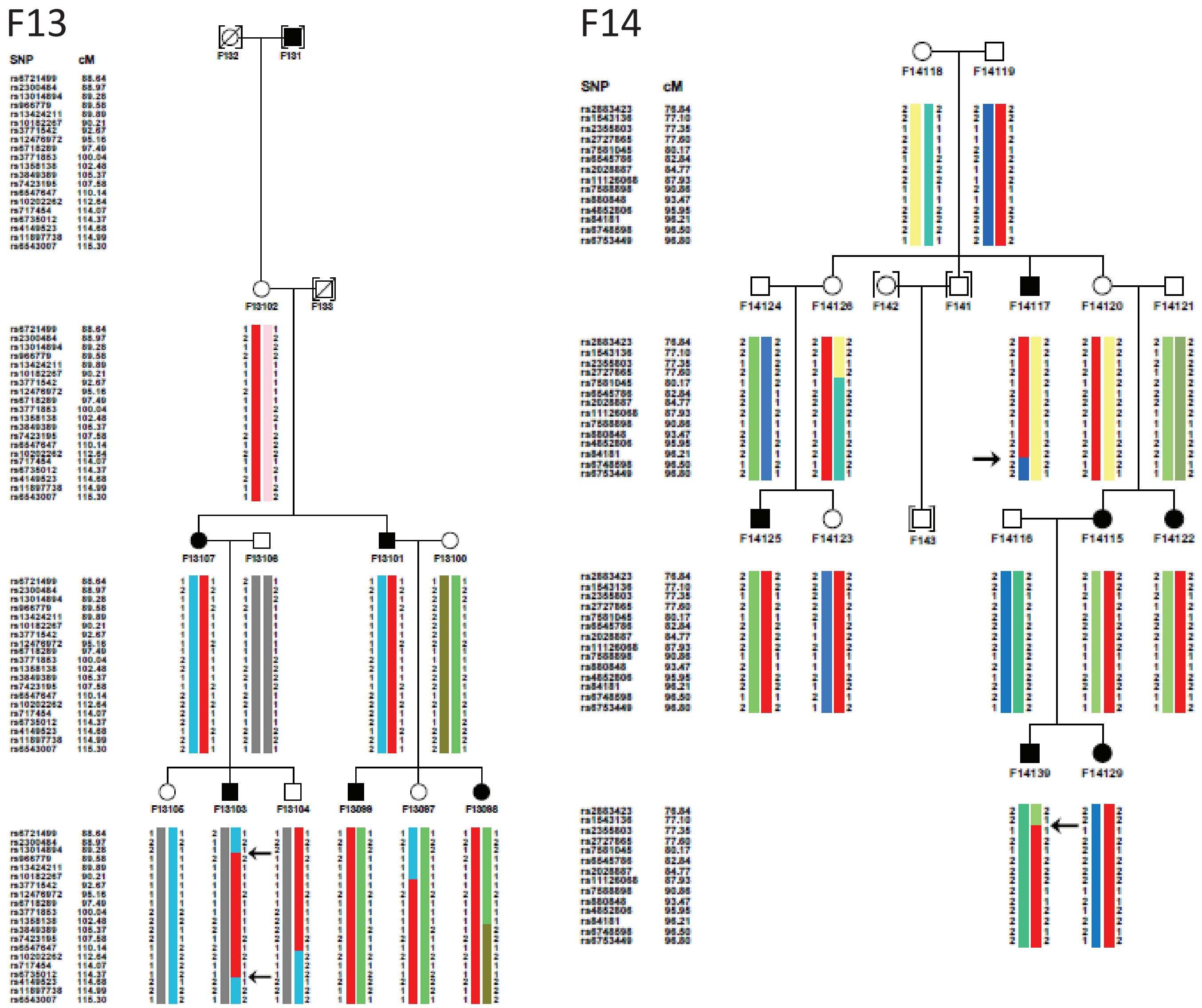

S6 Fig. Haplotype segregation in F13, locus 2p14-2q11.2; F14, locus 2p16.3-2p13.3. 20 SNPs (F13) or 14 SNPs (F14) illustrating haplotypes shared by all affected family members (SNPs do not depict exact locus boundaries; for precise values, see Table 1). Square = male; circle = female; black = affected; clear = unaffected; grey = unknown affection status; diagonal dash = deceased; symbols in brackets = no DNA available; red bar = segregating haplotype; 1 = major allele; 2 = minor allele; 0 = no DNA; arrows = approximate boundaries of familial locus; SNP = single nucleotide polymorphism; cM = centimorgan.
